# Bringing numerous methods for expression and promoter analysis to a public cloud computing service

**DOI:** 10.1093/bioinformatics/btx692

**Published:** 2017-11-06

**Authors:** Krzysztof Polański, Bo Gao, Sam A Mason, Paul Brown, Sascha Ott, Katherine J Denby, David L Wild

**Affiliations:** 1 Department of Statistics, University of Warwick, Coventry, UK; 2 Department of Mathematics, University of Warwick, Coventry, UK; 3 Systems Biology Centre, University of Warwick, Coventry, UK; 4 Department of Computer Science, University of Warwick, Coventry, UK; 5 Department of Biology, University of York, York, UK

## Abstract

**Summary:**

Every year, a large number of novel algorithms are introduced to the scientific community for a myriad of applications, but using these across different research groups is often troublesome, due to suboptimal implementations and specific dependency requirements. This does not have to be the case, as public cloud computing services can easily house tractable implementations within self-contained dependency environments, making the methods easily accessible to a wider public. We have taken 14 popular methods, the majority related to expression data or promoter analysis, developed these up to a good implementation standard and housed the tools in isolated Docker containers which we integrated into the CyVerse Discovery Environment, making these easily usable for a wide community as part of the CyVerse UK project.

**Availability and implementation:**

The integrated apps can be found at http://www.cyverse.org/discovery-environment, while the raw code is available at https://github.com/cyversewarwick and the corresponding Docker images are housed at https://hub.docker.com/r/cyversewarwick/.

**Supplementary information:**

Supplementary data are available at *Bioinformatics* online.

## 1 Introduction

Experimental techniques keep evolving at a great pace, constantly increasing the range of research questions that can be posed to the data. This creates a need for computational methods to keep up, be it with the decreasing cost of a laboratory procedure making larger scale experimental designs possible (Windram *et al.*, 2012) or due to an improved technique leading to a drastic change in the character of the resulting data (Anders and Huber, 2010). As such, a huge number of novel algorithms are being created, but their upkeep and usability vary greatly. Some methods take on the form of established, regularly updated packages with extensive documentation that are easy to set up and use locally (Gentleman *et al.*, 2004), whilst others are but a set of scripts attached to a research paper with no documentation or subsequent upkeep, quickly becoming very difficult to run. Another common issue among algorithms that do not take on the form of dedicated software packages is the quality of the implementation, with the scripts often created in programmer-friendly environments, leading to less efficient implementations (Penfold *et al.*, 2012; Polanski *et al.*, 2014). These factors make the application of a number of very useful methods much more challenging than it has to be. Over the years, a number of freely accessible cloud computing services have been made available to the scientific community for data analysis purposes. Examples include iPlant, now rebranded to CyVerse (Goff *et al.*, 2011) and Galaxy (Hillman-Jackson *et al.*, 2012). CyVerse is a National Science Foundation (NSF)-funded cyberinfrastructure that democratises access to data storage space, HPC and cloud computing facilities. CyVerse provides three key services to its users: the cloud-based Data Store that enables scientists to store and share very large datasets; the user-friendly Discovery Environment, in which they can work individually or collaboratively to analyze data using ‘apps’ built by the individual researchers or the wider community; and Atmosphere, through which users can access on-demand high-performance cloud computing power. To help spread effort, expertise and resources, CyVerse operates a distributed model within the US between TACC, Cold Spring Harbor Laboratory and the University of Arizona, and its platform has been designed with extension and replication in mind. CyVerse middelware (computational software interfaces between services) enables integration of multiple data sources and HPC facilities to provide one simple user interface. Since its launch in 2008, more than 1800 researchers now use CyVerse. Such platforms make it possible to outsource the large computational burden of analysing big data. A large number of algorithms present on a single server create the need for separate environments to avoid dependency clashes, leading to the use of technologies such as Docker (Merkel, 2014). CyVerse UK is a joint effort between the Universities of Warwick, Liverpool and Nottingham and the Earlham Institute, to create a UK node of the CyVerse collaborative. Here we document the result of a large body of work that has been carried out at the University of Warwick as part of the CyVerse UK initiative and made 14 popular tools much more available and easy to use.

## 2 Tool selection

Readily available tools on large cloud computing services predominantly center around high-throughput sequencing data analyses. Tools for complex analyses of expression data and regulatory sequences are underrepresented, while often requiring a large computational overhead in the case of more complex methodology. As such, a number of previously published, locally created methods were selected to produce a well-rounded time course expression data analysis package for inclusion into CyVerse. Differential expression can be handled with GP2S (Stegle *et al.*, 2010) in a typical control-treated scenario, or the gradient tool (Breeze *et al.*, 2011) to identify timing of first change in single condition datasets. Clustering can be performed with BHC (Cooke *et al.*, 2011) for a Bayesian hierarchical approach, TCAP (Kiddle *et al.*, 2010) to obtain complex regulatory modules with a rich information metric capable of capturing inversions and time shifts, or Wigwams (Polanski *et al.*, 2014) for co-regulation across subsets of multiple datasets. Transcription factor binding site overrepresentation of the resulting gene groups can be done with known sequences using the hypergeometric motif test (Breeze *et al.*, 2011) or *de novo* with MEME-LaB (Brown *et al.*, 2013), while a BiNGO-friendly (Maere *et al.*, 2005) output is created for GO term overrepresentation analyses to be carried out locally in Cytoscape. Causal network inference can be performed using CSI (Penfold and Wild, 2011), as well as its extensions to handle multiple datasets in a hierarchical structure (Penfold *et al.*, 2012) or across multiple species (Penfold *et al.*, 2015). The flow of the analysis of a time course dataset with the provided tools is shown in panel A of Supplementary Figure S1. Other algorithms related to gene expression analysis have also been integrated: the reverse best hit orthologue detection and conserved promoter functionality of the APPLES suite (Baxter *et al.*, 2012), as well as footprint identification in DNase-seq data by Wellington (Piper *et al.*, 2013) along with its differential analysis extension Wellington-bootstrap (Piper *et al.*, 2015).

## 3 Deployment standards

The first step in the preparation of each tool was to create efficient, stable implementations in freely available programming languages where this was not already the case. As such, the algorithms previously only available in Matlab (gradient tool, TCAP, Wigwams, CSI, hCSI, oCSI) were re-coded into Python, greatly decreasing their run time. Other algorithms which were previously available outside of Matlab (GP2S, hypergeometric motif test, APPLES) had their code bases refined to increase stability and user friendliness. Once implementations of adequate quality were available, the algorithms were housed in standalone Docker containers (Merkel, 2014). This makes the methods future-proof, with dependency problems solved proactively by encapsulating functional versions of the programs. This has already proven advantageous, with GP2S requiring a very particular Python 2.7 setup to function properly. The resulting Docker containers were imported into the CyVerse Discovery Environment and graphical, user-friendly apps were created. These can all be found by searching for ‘uk cyverse’ in the app window, and the algorithms all run remotely on CyVerse hardware. Each app links to exhaustive documentation and a set of test data, detailing in great depth how to format the input and what each available parameter is responsible for. The output is created with user friendliness in mind, and usually features some form of visualization, often as interactive webapps. Excerpts of visual output produced by the programs can be seen in panels B and C of Supplementary Figure S1. The complete results of each analysis get compressed into a single archive for ease of downloading to a local machine and further investigation. A tutorial on using the tools in the time course expression data pipeline and chaining these together using helper apps has also been created, and can be accessed at https://github.com/cyversewarwick/expression_tutorial. Every stage of the development process detailed above is publicly available—the raw code is housed on GitHub, which is chained to a DockerHub account, automatically rebuilding individual repositories when they get updated, with the newest versions of the images being used by CyVerse apps.

## Funding

This work has been supported by the Biotechnology and Biological Sciences Research Council grant BB/M018431/1.

*Conflict of Interest*: none declared.

## Supplementary Material

Supplementary Data
