# Supplementary material for: Bringing numerous methods for expression and promoter analysis to a public cloud computing service
Source: Bioinformatics. 2017 Nov 6;34(5):884–6. doi: 10.1093/bioinformatics/btx692 (PMC6030968; doi:10.1093/bioinformatics/btx692)
Supplement: Supplementary Data [file btx692_supp.zip › btx692_suppl-data/Supplementary.pdf]

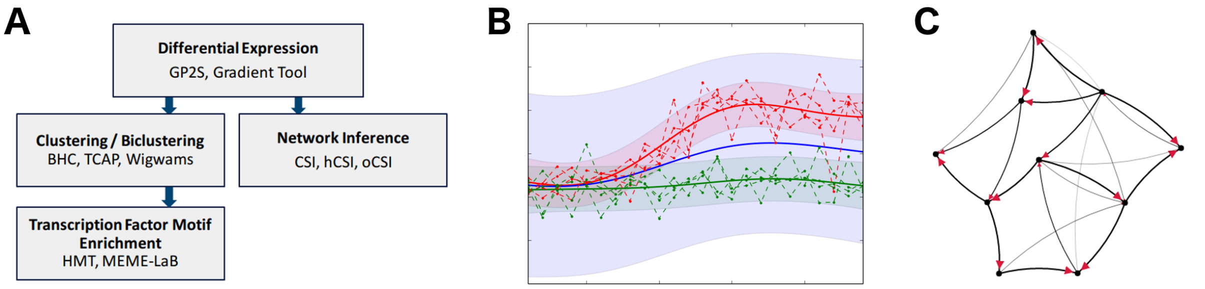

Figure 1: **A:** Workflow of a typical time course data analysis facilitated by some of the tools provided; **B:** a visualisation of GP2S model fits for expression data in two conditions (red, green) compared to an alternative, non-differential model (blue); **C:** a network visualisation in the CSI result webapp. Thickness of edges represents confidence in interactions.
