# Supplementary figures and images for: Bringing numerous methods for expression and promoter analysis to a public cloud computing service
Source: Bioinformatics. 2017 Nov 6;34(5):884–6. doi: 10.1093/bioinformatics/btx692 (PMC6030968; doi:10.1093/bioinformatics/btx692)

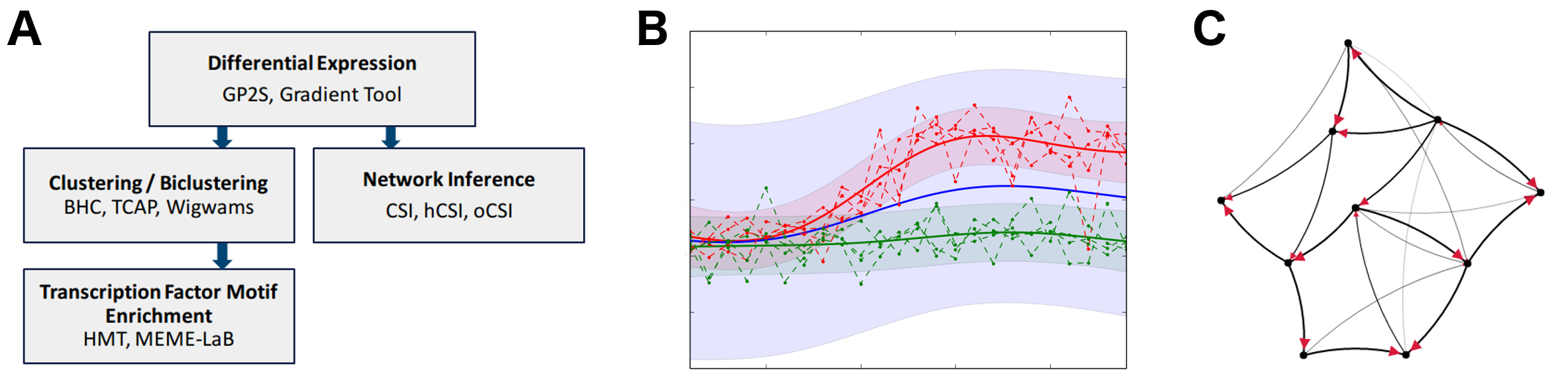

Supplement: Supplementary Data [file btx692_supp.zip › btx692_suppl-data/figure.tiff]
